# RETRACTION: Surgical Site Wound Infection and Pain After Laparoscopic Repeat Hepatectomy for Recurrent Hepatocellular Carcinoma

**DOI:** 10.1111/iwj.70301

**Published:** 2025-03-06

## Abstract

**RETRACTION:** WangX.‐B. , LinJ.‐M. , ChenY.‐P. , and YeX.‐X. , “Surgical Site Wound Infection and Pain after Laparoscopic Repeat Hepatectomy for Recurrent Hepatocellular Carcinoma,” International Wound Journal 20, no. 8 (2023): 3262–3270, 10.1111/iwj.14206.37086085 PMC10502282

The above article, published online on 22 April 2023, in Wiley Online Library (http://onlinelibrary.wiley.com/), has been retracted by agreement between the journal Editor in Chief, Professor Keith Harding; and John Wiley & Sons Ltd. Following an investigation by the publisher, all parties have concluded that this article was accepted solely on the basis of a compromised peer review process. The editors have therefore decided to retract the article. The authors did not respond to our notice regarding the retraction.

**RETRACTION:** X.‐B. Wang , J.‐M. Lin , Y.‐P. Chen , and X.‐X. Ye , “Surgical Site Wound Infection and Pain after Laparoscopic Repeat Hepatectomy for Recurrent Hepatocellular Carcinoma,” International Wound Journal 20, no. 8 (2023): 3262–3270, 10.1111/iwj.14206.37086085 PMC10502282

The above article, published online on 22 April 2023, in Wiley Online Library (http://onlinelibrary.wiley.com/), has been retracted by agreement between the journal Editor in Chief, Professor Keith Harding; and John Wiley & Sons Ltd. Following an investigation by the publisher, all parties have concluded that this article was accepted solely on the basis of a compromised peer review process. The editors have therefore decided to retract the article. The authors did not respond to our notice regarding the retraction.

